# Non‐invasive monitoring of multiple wildlife health factors by fecal microbiome analysis

**DOI:** 10.1002/ece3.8564

**Published:** 2022-02-09

**Authors:** Samuel B. Pannoni, Kelly M. Proffitt, William E. Holben

**Affiliations:** ^1^ Franke College of Forestry and Conservation University of Montana Missoula Montana USA; ^2^ Montana Fish Wildlife and Parks Bozeman Montana USA; ^3^ Cellular, Molecular and Microbial Biology Program University of Montana Missoula Montana USA

**Keywords:** 16S‐rRNA survey, conservation, host‐microbiome, machine‐learning, microbial‐biogeography, non‐invasive, wildlife

## Abstract

Fecal microbial biomarkers represent a less invasive alternative for acquiring information on wildlife populations than many traditional sampling methodologies. Our goal was to evaluate linkages between fecal microbiome communities in Rocky Mountain elk (*Cervus canadensis*) and four host factors including sex, age, population, and physical condition (body‐fat). We paired a feature‐selection algorithm with an LDA‐classifier trained on elk differential bacterial abundance (16S‐rRNA amplicon survey) to predict host health factors from 104 elk microbiomes across four elk populations. We validated the accuracy of the various classifier predictions with leave‐one‐out cross‐validation using known measurements. We demonstrate that the elk fecal microbiome can predict the four host factors tested. Our results show that elk microbiomes respond to both the strong extrinsic factor of biogeography and simultaneously occurring, but more subtle, intrinsic forces of individual body‐fat, sex, and age‐class. Thus, we have developed and described herein a generalizable approach to disentangle microbiome responses attributed to multiple host factors of varying strength from the same bacterial sequence data set. Wildlife conservation and management presents many challenges, but we demonstrate that non‐invasive microbiome surveys from scat samples can provide alternative options for wildlife population monitoring. We believe that, with further validation, this method could be broadly applicable in other species and potentially predict other measurements. Our study can help guide the future development of microbiome‐based monitoring of wildlife populations and supports hypothetical expectations found in host‐microbiome theory.

## Graphical abstract

Our goal was to evaluate linkages between non‐invasive fecal microbiome communities in Rocky Mountain elk (*Cervus canadensis*) and four host factors including sex, age, population, and physical condition (body‐fat). Our results show that elk microbiomes respond to both the strong extrinsic factor of biogeography and simultaneously occurring, but more subtle, intrinsic forces of individual body‐fat, sex, and age‐class. Our findings help unite some formerly unconfirmed expectations regarding multiple host factors in host‐microbiome theory. The products of such efforts could eventually provide insights and novel solutions to current wildlife management issues and allow threatened and endangered species to be studied with less perturbation.

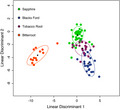

## 1 INTRODUCTION

Plummeting global biodiversity has created an overwhelming demand for improved species conservation (Pimm et al., 2014). Lacking the required funding to meet increasing conservation demands, managers and conservationists have necessarily adopted more spartan conservation strategies to stretch their limited personnel and budgets, analogous to medical “*triage*” and “*hospice*” (Gameiro et al., 2020; Peterson et al., 2020). These conservation strategies seemingly are stopgap measures, since the numerous anthropogenic forces that erode the sustainability of wildlife populations still loom large (Ceballos et al., 2015).

Non‐invasive monitoring methods can support conservation efforts by helping to fill gaps in knowledge vital to protecting and managing species of concern. These methods have been rapidly gaining traction in applied wildlife conservation and are becoming more cost‐effective thanks to breakthroughs in molecular and genomic technologies (Carroll et al., 2018). One such method is fecal microbiome analysis, which may help to strike a balance between the competing themes of hyper‐efficient management needs and costly monitoring data, while also meeting public expectations for animal welfare (Dubois & Harshaw, 2013).

Metagenomics and other culture‐independent techniques for studying host‐microbiomes have begun to provide insights relevant to non‐invasive wildlife conservation (Bahrndorff et al., 2016; Redford et al., 2012; Trevelline et al., 2019; West et al., 2019). Numerous studies have established that microbiomes reflect and sometimes dictate interactions connecting the host organism with its environment, which impacts host fitness (Zilber‐Rosenberg & Rosenberg, 2008). The host and its microbial complement are co‐evolving entities that embody a single evolutionary unit that selection acts upon, leading to the emerging “holobiont” concept (Bordenstein & Theis, 2015). The relevance of host microbiomes to conservation efforts resides in the numerous opportunities to obtain conservation‐relevant information from wildlife bacterial communities using non‐invasive fecal samples (Lynch & Hsiao, 2019).

Non‐invasive approaches are quickly becoming vital components in modern conservation practice for both practical reasons and due to their resonance with public sensitivities about animal welfare (Allendorf et al., 2010; Dubois & Harshaw, 2013). In addition to helping to meet changing public priorities for animal welfare in conservation, the practical value of non‐invasive methods includes relatively low‐cost, low‐effort sampling (i.e., of feces, feathers, hair, urine, saliva, etc.) for estimation of individual and population statistics, often with improved performance and sample sizes relative to more invasive methods of gathering data (Allendorf et al., 2010).

In addition to already established non‐invasive methods, host‐microbiome associations may overlap with, or provide access to, additional estimates relevant for wildlife conservation (Redford et al., 2012). Specifically, microbiome findings have expanded on this overlap to describe finer‐scale associations between microbiomes and host diet, sex, stress, age, disease, physical condition, and biogeography (Zhu, Wang, & Bahrndorff, 2021). Alongside other non‐invasive sample sources and methods such as analyzing hair snags or feathers, microbiome studies intersect promisingly with the needs of conservation practitioners. Microbiomes can provide an additional layer of information about animal condition, senescence, stress, and disease that may become useful for monitoring wild and captive populations in the future (Youngblut et al., 2019).

To investigate the potential of the microbiome for applied conservation and monitoring, we sought to further develop and apply bioinformatic tools to associate ingesta‐free body‐fat percentage (hereafter body‐fat), age, sex, and biogeography, with the fecal microbiomes from four populations of Rocky Mountain elk (*Cervus canadensis*). Although different host‐microbiome associations are represented in the literature, it is rare for multiple conservation‐relevant microbiome associations to be tested across multiple populations within a single study species, as was done here.

Elk are large mammalian ungulates in the Family *Cervidae*. Six subspecies of elk thrived across most of North America prior to European settlement, after which overexploitation and habitat loss severely bottle‐necked population numbers, causing extinction in two sub‐species (Anderson, 1972; Toweill & Thomas, 2002). Elk are once again broadly distributed thanks to active management, with reintroductions helping to repopulate their native range in the eastern United States, hereafter US (Popp et al., 2014). Wherever elk occur in North America, they have earned an iconic status among big game hunters (Popp et al., 2014). Elk are managed to maintain stable populations despite significant variation in annual harvest rates that can exceed 80 percent (Slabach et al., 2018). Elk management, particularly in the western US, is further complicated by population‐specific instances of overpopulation, property damage, and disease spread (Higgins et al., 2012; Proffitt et al., 2011).

Elk and other wild ungulates are an integral part of ecosystems and can provide the majority of biomass in non‐human predator diets (Metz et al., 2012; Stewart et al., 2006). The importance of elk management is thus both ecological and economic. Elk management success depends on developing and deploying efficient means of population‐specific management, especially considering that competing interests and stakeholder groups (e.g., hunters vs. non‐consumptive users) often require geographically specific outcomes. Development of non‐invasive methods to produce population‐specific monitoring data is a natural response to this challenge.

In the current study, we performed a survey of elk fecal bacterial microbiomes using 16S rRNA gene sequencing across four discrete Montana populations. Microbial communities are complex, with hundreds to thousands of interacting species (Boon et al., 2014). Both host and environmental factors influence community composition, with community differences often driven by a subset of taxa (Morris et al., 2012). Discovering individual taxa or subsets of microbes that are consistently associated with difficult‐to‐measure factors in elk could let those taxa be non‐invasive proxies for those factors. To test whether individual elk data could be correlated with non‐invasive fecal microbiome data, we used a feature selection (FS) algorithm to select the most informative microbes, and then used linear discriminate analysis classifiers (LDA) with cross‐validation (CV) (similar to Liu et al., 2014) to test whether this reduced microbiome could predict population, age, body‐condition, and sex.

## 2 METHODS

### 2.1 Field collection of fecal samples and physiological measurements

**FIGURE 1:**
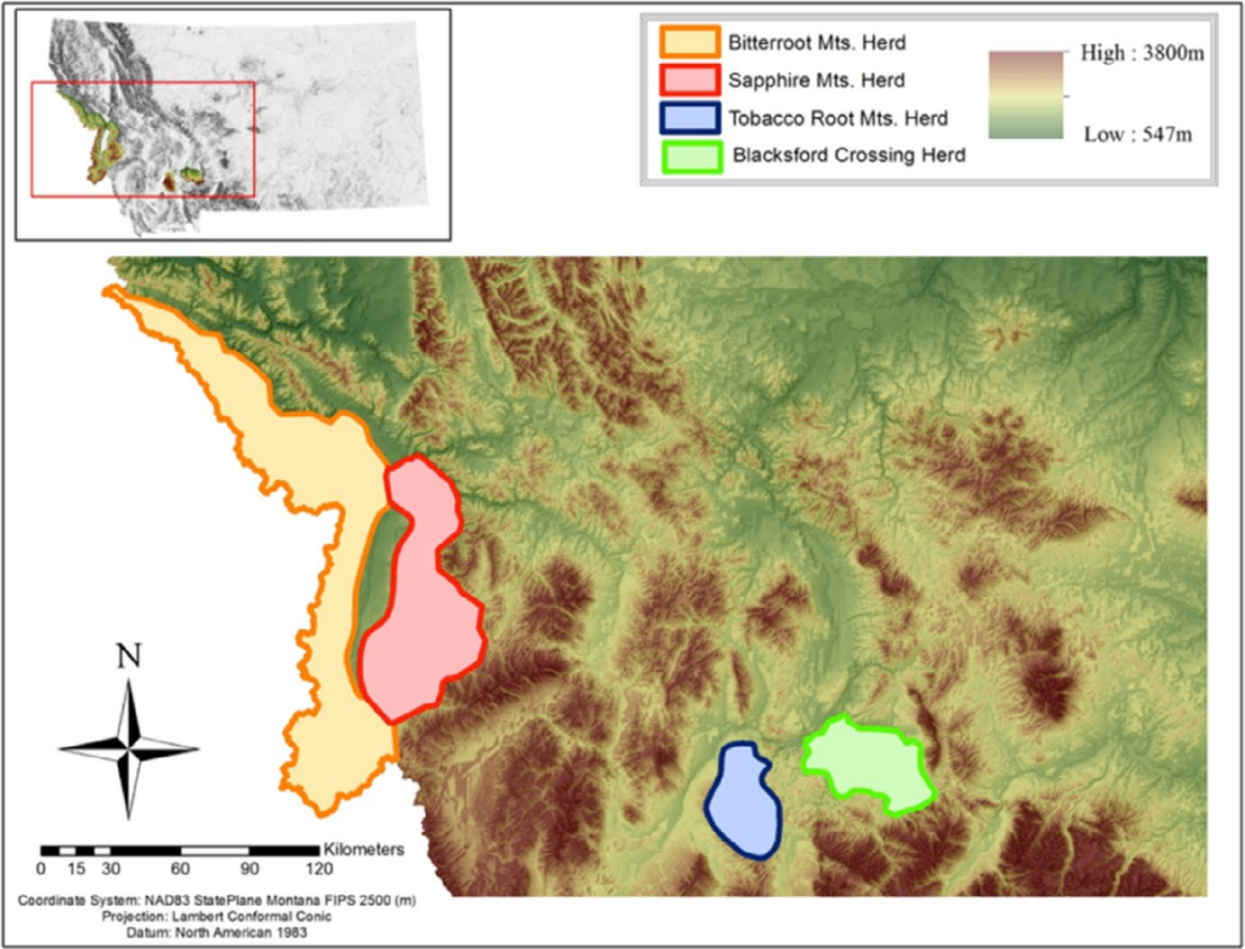
Sampling location map. Map of the Montana study area. Four elk populations were sampled in 2014 as indicated by colored polygons

Collection of scat samples (*n* = 110), sex determination (*n* = 104), age data (*n* = 34), body‐condition data (*n* = 73), and radio‐collaring of elk were conducted in February 2014 by Montana Fish Wildlife and Parks (MT FWP) during a helicopter capture operation. We received fecal pellet samples and physiological measurements for elk from four populations: Bitterroot Mountains (*n* = 13), Sapphire Mountains (*n* = 54), Black's Ford area of the Madison River (*n* = 25), and Tobacco Root Mountains (*n* = 18) (Figure 1). Animal capture and sampling were conducted using currently available and accepted methods for animal capture and restraint, body‐fat estimates, sex classification, and age (Cook et al., 2001, 2010). Elk sex was observed and recorded in the field during captures (87 females, 17 males) but sex was not recorded for all individuals, thus these elk of unknown sex were filtered from sex‐specific statistical comparisons (*n* = 104). Body condition data were collected in females from Sapphire Mountains, Black's Ford, and Tobacco Root Mountains (*n* = 73) during capture using a portable ultrasound machine to estimate levels of ingesta‐free body‐fat (Cook et al., 2001, 2010). Body‐fat data was not collected for males since these measurements are not informative for bull elk (Cook, 2002). Age information was collected by observing tooth eruption and wear patterns for a subset of female elk in the Sapphire Mountains population (Hamlin et al., 2000). Fecal samples were aseptically collected for all elk with a cleanly gloved hand and placed in whirl‐pak sample bags. Samples were placed on wet ice in a cooler in the field, then frozen at −20°C after each sampling day prior to delivery on wet ice to the lab.

### 2.2 Sample preparation, DNA extraction, and sequencing

Frozen elk fecal pellets (−20°C) were prepared for DNA extractions by separating a standard weight (250 mg) from one randomly selected pellet per individual using a sterile petri dish (10 cm) and sterile safety razor blade for each sample. This fraction was placed into the designated sample tube from the Qiagen PowerSoil DNA extraction kit (Qiagen Inc., Germantown, MD) and processed using the manufacturer's recommended protocol. The resulting purified metagenomic DNA was eluted with 100 µl PCR‐grade water and stored at −20°C prior to further analysis.

To assess the bacterial community present in the fecal DNA extraction, we used a generally conserved (i.e., “universal”) 16S/18S barcoded primer set (536F and 907R) and PCR designed for the V4 and V5 variable regions of the rRNA gene (Holben et al., 2004) using 1μl of elk fecal sample metagenomic DNA standardized to 25 ng/μl. Once amplified, the resulting amplicons were size‐fractionated on 1.5% agarose gels, with 16S bacterial amplicons excised and gel purified using the QIAGEN Gel Purification kit (QIAGEN, Germantown, MD) following the manufacturer's recommended protocol for downstream direct sequencing. An Illumina MiSeq platform (San Diego, CA, USA) was used to obtain 300 base‐pair (bp) paired‐end sequencing using the Illumina MiSeq Reagent Kit.

### 2.3 Sequence analysis

Primer sequences were removed using Cutadapt software and any reads without a mate‐pair or recognizable primer sequence were discarded (Martin, 2011). Read quality was summarized visually with FastQC and MultiQC (Andrews, 2010; Ewels et al., 2016). The DADA2 package (Callahan et al., 2016) was used in the R environment to further quality filter and trim paired reads according to the published workflow (Callahan, 2021). The remaining sequences were denoised and dereplicated. We next identified amplicon sequence variants (ASVs) from the resulting high‐quality sequences, merged forward and reverse reads, and removed chimeric sequences. ASVs were taxonomically assigned with the DADA2 instance of the Naïve‐Bayes classifier and the Ribosomal Database Project II release (Cole et al., 2009). A matrix was produced containing counts corresponding to the abundance of each ASV present in each elk sample and an additional paired matrix with each ASV’s taxonomic classification.

The Phyloseq R package (McMurdie & Holmes, 2013) was used to further filter and summarize ASV tables, measure sample, and group diversities and perform statistical analyses. A small number of non‐bacterial ASVs that belonged to Kingdom Archaea or in the Chloroplast Class were removed from this bacterial analysis. ASVs assigned to unknown phyla were removed since these are not informative for our analyses. All ASVs that failed phylogenetic assignment at the genus level were standardized to “g_unknown” in the taxonomy matrix and retained for diversity analyses. A separate data set with unknown genera removed was produced for use with FS‐LDA classification. We chose genus‐level classification for our analysis to balance the specificity of phylogenetic assignment with missing classifications (caused by the typically low number of species‐level assignments) to best assess biological relevance in our classifiers.

Normalization methods are a topic of hot debate in the field of molecular ecology since sequencing effort (reads/sample) is decoupled from biological sampling effort. There is no consensus approach for normalizing the large variance in reads for ecological count data without tradeoffs, but some popular methods are known to be unreliable (e.g., rarefaction) due to a high false discovery rate (FDR) (Lin & Peddada, 2020). Although several data normalization techniques have been suggested (e.g., Compositional Data Analysis (CoDA), variance stabilizing technique (VST), Analysis of Compositions of Microbiomes with Bias Correction (ANCOM‐BC), and cumulative‐sum averaging), they all include counterproductive tradeoffs. Thus, richness, alpha‐diversity (Shannon), and accompanying statistical tests between groups were calculated without rarefying sample data. Richness at the genus level was calculated per sample with *breakaway*, a nonlinear regression model that provides standard error (SE) and p‐values (Willis & Bunge, 2015). The *betta* function in the *breakaway* package was used to test for differences in sample richness (observed and unobserved diversity) across populations. Alpha‐diversity (Shannon) was calculated per sample using the *DivNet* package, which incorporates diversity estimates with correction for incomplete sampling (Willis & Martin, 2020). The *betta* function was used to calculate the significance (p‐values) of the alpha‐diversity estimates.

Beta diversity comparisons and Adonis tests (PERMANOVA) were performed after adjusting log‐transformed abundances by sample fractions calculated with the ANCOM‐BC method (Lin & Peddada, 2020). We calculated Unifrac, Unweighted Unifrac, and Bray‐Curtis distances using the vegan R package (Oksanen et al., 2017). These distances were reduced and plotted using PCoA (and RDA for Bray‐Curtis). Adonis tests were performed on the distances between covariates (999 permutations) using the Phyloseq R package (McMurdie & Holmes, 2013). Permutation tests for homogeneity of multivariate dispersions were conducted on each of the three distances (999 permutations) to indicate applicability of the Adonis test for each distance matrix.

### 2.4 Feature selection and cross‐validation

In microbiomes, not all taxa are associated with specific conservation‐relevant measurements in the host because some genera or species may perform a different unrelated, often unknown, function (Sharpton, 2018). The complete microbiome is thus assumed to contain “noisy” genera that sometimes obscure the biological patterns and associations present. We used a form of the Sequential Forward Floating Search algorithm (i.e., Feature Selection—FS; Pudil et al., 1994) to select for informative genera from the elk microbiome. This algorithm selects a subset of genera from the total pool using a heuristic method that maintains or improves the performance of the complete data set. FS avoids nesting issues where features (in this case, bacterial taxa) are falsely fixed early in the selection process creating a local maxima, which is an issue with other feature selection methods that results in reduced performance (Pudil et al., 1994; Saeys et al., 2007). By allowing all features to be added or subtracted as the algorithm progresses (essentially “floating” the selections), features interact to produce dynamic and unbiased performance results that are not dependent on starting conditions. The FS algorithm employed herein uses J3 scores, a form of scatter matrices that rewards close clustering within groups of data points and rewards increased distance between groups of data points using Euclidean distances in multidimensional space (Fukunaga, 1990). We produced feature tables of FS genera sequentially with 2 through 30 features (one table of FS genera for each number of features in the range).

For each set of genera in a feature table, a linear discriminate classifier was created and tested on the underlying count data with sample cross‐validation (CV), which uses a leave‐one‐out method of training and testing to reduce over‐fitting of the model to the training data set (Liu et al., 2014; Saeys et al., 2007). This method removes a sample (an individual elk's microbiome subset) from the training data, builds the model with the remaining samples (N‐1) then tries to predict the classification of the removed sample. This leave‐one‐out method is iterated across all samples (N) to calculate the CV accuracy.

We balanced classifier performance and over‐fitting by comparing the CV performance differences between multiple numbers of features for signs of overfitting (Braga‐Neto & Dougherty, 2004). Variance in model accuracies at each possible feature number (2 through 30) was obtained by running models with progressively zero pruned data sets from 0% ‐ 16% at each number of features, which also helped to assess model sensitivity. To help choose the optimal number of features for the final LDA visualizations, we generated a three‐dimensional Pareto front (Hwang et al., 1979) for multi‐parameter optimization including accuracy, number of features, and variance (not shown). Any set of features selected on the Pareto front was thus optimized for these parameters and had impartial support. We visualized this relationship with box plots of accuracy at different numbers of features (Figure S1).

### 2.5 Classifiers for each elk factor

Each classifier varied somewhat according to the desired factor to be predicted (i.e., biogeography, body‐fat, sex, or age). In some cases, multiple models were built to explore the results of different binning criteria on continuous data types (i.e., body‐fat and age). In the simplest case of categorical classification, we predicted population using the four available sampling locations known for all samples, including Bitterroot Mountains, Sapphire Mountains, Black's Ford area (Madison River), and the Tobacco Root Mountains. Similarly, categorical sex classification was done using adult male and female elk across the four Montana populations (*n* = 104 after filtering). Due to female elk being more abundant and prioritized during sampling, the sample data was skewed toward females (87:17). To provide equally weighted classifier training groups we chose to normalize bin sizes between males and females both by sampling males with replacement (bootstrapping) up to 87 samples or by randomly rarefying female samples to 19 individuals (not shown). Three random bootstrap iterations were performed in each case to determine the sensitivity of sampling individuals with replacement or rarefaction.

To classify elk factors with continuous data types (i.e., body‐fat and age) we chose to use three different models for predicting body‐fat in female elk from three populations (Bitterroot not sampled) and a single binary model for age. The body‐fat models had 2, 3, or 4 body‐fat classes (only the 4 class model shown; 2 and 3 not shown). These classes were chosen to provide balance in bin sizes for classifier training and less for biological significance. The bin cutoff values for body‐fat were: 2‐class model >8% or <8%, 3‐class model >7%, 7–9%, and <9%, and lastly the 4‐class model >7%, 7–8%, 8–9%, and <9%. We chose to use a two‐class model for female elk age: early reproductive age (3–6 years) and prime reproductive age (7–10) females. We chose 2 age bins based on the available female age data and prioritized balanced bin sizes for age ranges.

## 3 RESULTS

### 3.1 Study populations and field measurements

Sampling and sequencing included 110 animals, but due to incomplete sexing data, only 87 females and 17 males (*n* = 104) are represented in the analyses with female ages ranging from 3 to 10 years (sample mean 6.38). Female elk body‐fat ranged from 5.70% to 13.29% (sample mean 7.95%). The results of the field measurements taken are summarized in Table S1.

### 3.2 Sequencing and quality control

Sequencing and QA/QC sequence processing of partial 16S rRNA amplicons from each fecal sample provided a total of 8,774,731 paired‐end sequences (forward and reverse reads) across all elk sampled, with sample depths varying from 5571 to 128,069 sequences per individual (mean depth 79,770). Primers were trimmed from paired‐end sequences with filtering parameters that required a read to have a primer present, a minimum length of 100 bp, and a mate‐pair, which resulted in 8,690,840 sequences remaining. Filtering and trimming on quality (max of 2 errors, no Ns, truncate at the instance of quality = 2) and length (minimum of 80 bp after quality trimming) reduced the number of sequences retained to 7,599,271. The remaining sequences were dereplicated and amplicon sequence variants (ASVs) were inferred independently with forward and reverse reads (by sequencing run) using the DADA2 error model. Forward and reverse reads were merged into single reads (4,374,192 remaining), sequencing runs were combined, and chimeric sequences were removed, resulting in 3,386,467 high‐quality paired sequences remaining. Read depths per animal ranged from 1,103 to 66,124 (mean = 30,786; four samples below 1500 reads) after these quality filters. Sequence filtering results are summarized in Table S2.

### 3.3 16S gene survey of elk ASVs

Sequencing of partial 16S rRNA amplicons from each fecal sample provided a survey of bacterial presence and abundance in the elk fecal microbiome. ASV counts per animal ranged from 1008 to 65,894 after quality filtering with a mean count of 30,736. After filtering, a total of 11,957 unique ASVs were indicated across all samples. The distribution of unique ASVs (observed richness) within individual samples ranged from 30 to 1290, with a mean of 714.8 unique ASVs per individual (see Table S2). After assigning phylogeny to ASVs, there were 136 unique genera. The top 10 most abundant ASVs across all samples (after VST) were classified to the genera *Sporobacter*, *Bacteroides*, *Phascolarctobacterium*, or "g(enus)__Unknown", all within the phyla Bacteroidetes, Firmicutes or Proteobacteria (For bacterial genera proportional abundance see Figure S2).

Richness at the genus level was calculated using *breakaway* richness (Willis & Bunge, 2015) and is summarized in Figure S3. Global *breakaway* richness for the elk in this study, defined as the total diversity including the unobserved (unsampled) count, was estimated at 461 genera (compared to the 136 genera observed). All populations varied significantly (*p* ≤ .01) in richness from the Bitterroot population (*breakaway* richness estimate intercept 866.2), except the Tobacco‐Root population (*p* = .082). The *breakaway* Shannon diversity estimate was calculated for each sample (Figure S4) and similar to richness, was significantly different (*p* ≤ .001) between all populations, except for the Tobacco‐Root population (*p* = .255).

Beta‐diversity, the diversity between bacterial communities of individual elk was calculated between samples on the abundance‐adjusted dataset using Unifrac, unweighted‐Unifrac, and Bray‐Curtis distances. The resulting PCoA and RDA plots show clustering at the population level (Figure S5). When comparing between elk populations with Adonis tests, bacterial community distances before and after feature selection were more similar within elk populations than between populations, and mean distances between the populations were significant (*p* = .001) for all distances. Elk sex was included as a covariate in all Adonis models and was significant for Bray‐Curtis (*p* = .002) and weighted‐Unifrac (*p* = .018) but not for unweighted‐Unifrac (*p* = .266). Similarly, permutation tests on beta dispersions were significant (*p* = .001) for Bray‐Curtis and weighted‐Unifrac, but lower for unweighted‐Unifrac (*p* = .05).

### 3.4 Classifiers for elk ecological factors

**FIGURE 2:**
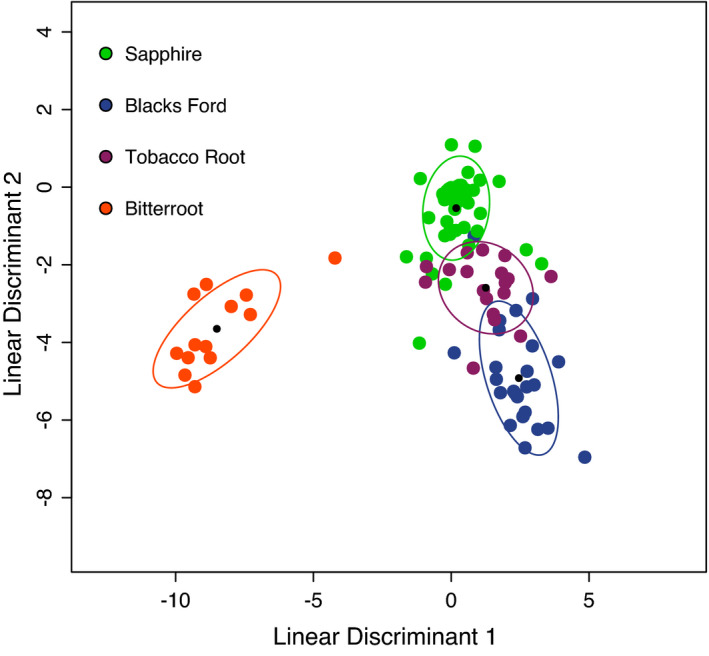
FS‐LDA ordination plot of female elk microbiome samples from 4 populations in Montana as a function of geographic location. Colored circles represent different populations as indicated; black circles are the centroid of each cluster, and colored ellipses depict 1 standard deviation from the cluster centroid. This classifier was trained using leave‐one‐out cross‐validation, producing 81% model accuracy using 23 genera

Elk populations included in the biogeography classifier were the Bitterroot Mountains, Sapphire Mountains, Black's Ford area of the Madison River, and the Tobacco Root Mountains. Analysis with FS‐LDA (Figure 2) and PCoA of total elk bacterial microbiome data (Figure S5) showed strong patterns of elk population structure (biogeography) with the FS‐LDA method showing a tighter relationship. The elk FS‐LDA population classifier performed with 81% CV accuracy using 23 bacterial genera. Consistent accuracy values across all FS dimensions from 2 to 30 were observed (Figure S1).

**FIGURE 3:**
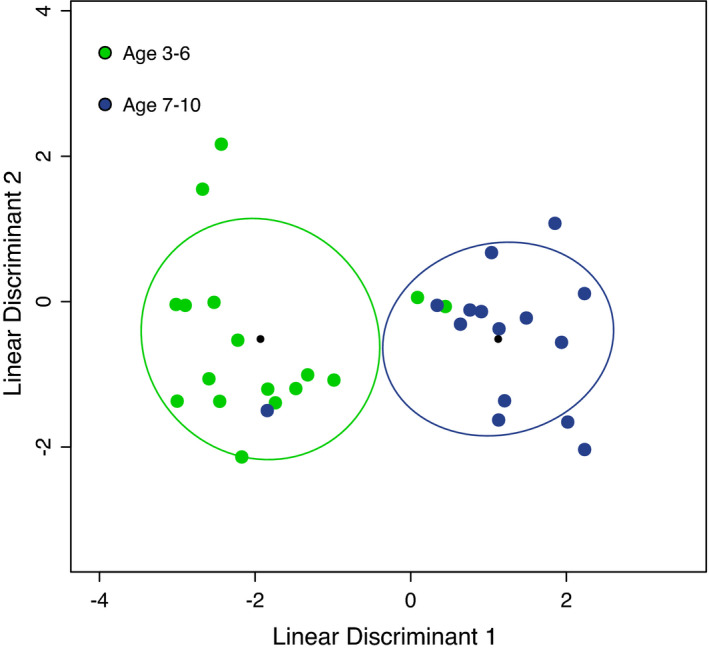
FS‐LDA ordination plots of female elk microbiomes from the Sapphire population as a function of age. Colored circles represent elk age group as indicated. Black circles are the centroid of each cluster, and ellipses depict 1 standard deviation from the centroid. The classifier was trained using leave‐one‐out cross‐validation, producing 87% model accuracy using 8 genera

Age estimates for female elk in the sapphire population (*N* = 34) ranged from 3 to 10 years. The model was trained on 2‐classes, female elk aged 3–6 years and 7–10 years. The model used 8 taxa and performed with 87% CV accuracy (Figure 3).

**FIGURE 4:**
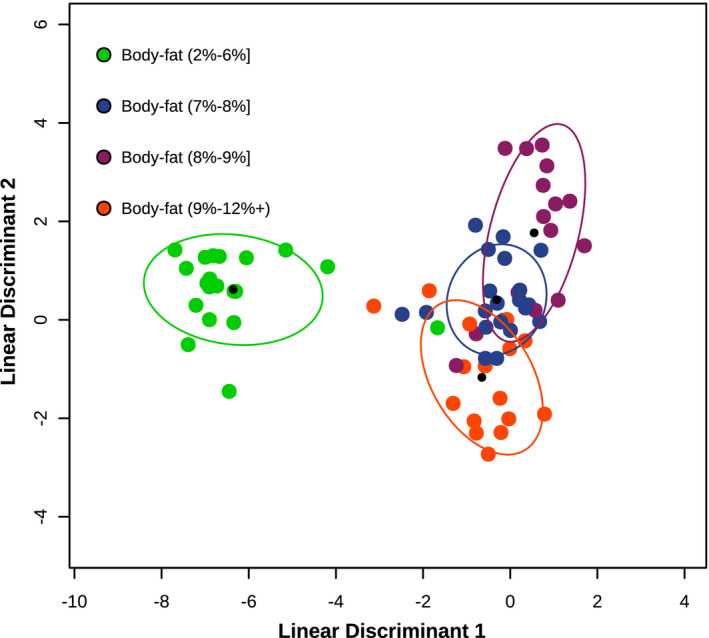
FS‐LDA ordination plot of female elk microbiomes (*n* = 73) from 3 populations as a function of ingesta‐free body‐fat (body‐fat). Filled circles are elk microbiomes predicted into ingesta‐free body‐fat categories including body‐fat below 7% (red), between 7 and 8% (green), between 8 and 9% (blue), and above 9% (orange). Black circles are the centroid of each cluster, and ellipses depict 1 standard deviation from the centroid. The classifier was trained using leave‐one‐out cross‐validation, producing 58.3% model accuracy with 28 genera

FS‐LDA was used to classify bacteria as a function of body‐fat from female elk across three populations and was trained using either two, three, or four classes of body‐fat (Figure 4, some data not shown). The performance of the classifier for two elk body‐fat categories (above or below 8% body‐fat) was supported by low levels of overlap between clusters and by high CV accuracy of 91% (where 50% is the expected null hypothesis). The three‐class model of body‐fat (below 7%, 7%–9%, and above 9%) was performed at 77.8% accuracy (compared to a 33% null) with 30 taxa. The four‐class model (body‐fat below 7%, 7%–8%, 8%–9%, and above 9%) was performed at 58.3% accuracy (25% null) with 28 taxa.

**FIGURE 5:**
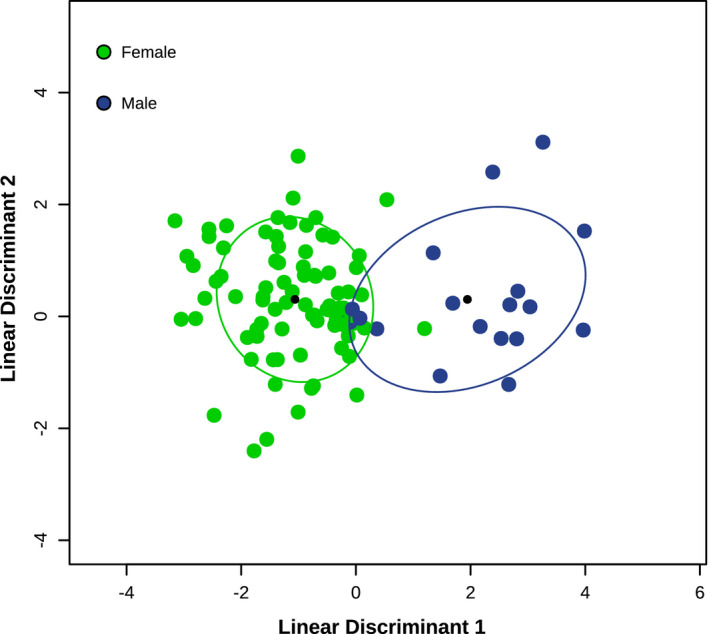
FS‐LDA ordination plots of elk microbiomes from 4 populations as a function of sex. Colored circles are elk microbiomes while colors represent elk sex: red = female and green = male. Black circles are the centroid of each cluster, and ellipses depict 1 standard deviation from the centroid. Nineteen male elk were bootstrapped (sampled with replacement) to *n* = 87 to provide equal weight to the classifier training groups. The classifier was trained using leave‐one‐out cross‐validation, producing 90% model accuracy with 24 genera

Classification of sex was obtained with high CV accuracy after normalizing bin sizes between males and females by either sampling males with replacement (bootstrapping) up to 87 samples (Figure 5, iterative data not shown), or by randomly rarefying female samples to 17 individuals (not shown). Bootstrapping produced ordination clustering by sex with 90% CV accuracy. Multiple random bootstrap iterations were performed with little change to the outcome of the classifier, indicating the classifier for sex was not sensitive to the random effects of sampling with replacement (some data not shown). Random rarefaction produced similar results in the visualization, with a comparable CV accuracy of 89%, which was qualitatively repeated across multiple random rarefaction iterations.

For a list of genera selected in each classifier see Table S3.

## 4 DISCUSSION

This study provides strong evidence for linkages between several elk physiological measurements and the host microbiome, as well as support for biogeographical structuring of the host microbiome. Analyses attempting to discover various individual factors influencing host‐microbiome structure in animals are common (Corl et al., 2020; Fountain‐Jones et al., 2020; Ren et al., 2017; Yuan et al., 2015), with previous research supporting both biogeographic structuring (Martiny et al., 2006; Moeller et al., 2017) and host‐associated responses (Cho & Blaser, 2012; Mshelia et al., 2018a). Despite a strong theoretical expectation, synergistic support for multiple intrinsic and extrinsic factors structuring host‐microbiomes is rarely demonstrated in a single study as demonstrated here.

The Montana elk intestinal bacterial microbiome is dominated by phyla Firmicutes, Bacteroidetes, and Proteobacteria, typical of ruminant animals that degrade complex carbohydrates anaerobically into volatile fatty acids (Henderson et al., 2015). Our findings for total fecal microbiome composition were largely consistent with the only other known surveys of North American elk microbiomes to date (Gruninger et al., 2014; Kim et al., 2019). Our deeper survey uncovered a large proportion of unclassified genera and species despite using current methods and databases, suggesting that this system is still ripe for future microbial description.

Our study discovered strong patterns of biogeography in four populations of elk using two mechanistically different approaches (PCoA and FS‐LDA). This suggests that environmental and site‐specific effects are important in structuring fecal microbiome communities within an individual's home range. Alpha and beta diversity results corroborate this by demonstrating that bacterial diversity is more similar among individuals within a population than between populations. Adonis tests between populations and *breakaway* measurements of richness and Shannon diversity were significant (*p* = .0001, *p* ≤ .01) between all populations except for the Tobacco‐Root population which had high variation among individuals (*p* = .255). Our results corroborate theoretical expectations and limited findings of spatially structured wildlife microbiomes, including the existence of endemic taxa, non‐random similarity across taxa from different landscape types, maintenance of host‐microbiome diversity by dispersal limitation in mammals, and biogeography as observed in the European house mouse (Linnenbrink et al., 2013; Martiny et al., 2006; Moeller et al., 2017).

Other studies have found environmental variables like temperature, salinity, and changes related to captivity to be important in determining microbiome species composition and distribution (Haworth et al., 2019; Kivistik et al., 2020; Zhu, Zhu, et al., 2021). However, comparisons from studies sampling multiple discrete populations of the same study species are rare (Linnenbrink et al., 2013). Prediction accuracies for our population classifier were consistently high across all FS dimensions tested (2 taxa through 30 taxa), indicating that the contribution of biogeography and its effects are widespread throughout the elk microbiome. Although not measured in this study, the biogeographic patterns found in the elk bacterial microbiome may be partly influenced by small‐scale differences in forage types and availability between the sampled populations of elk, since diet is known to influence gut microbiomes (Petri et al., 2013). Our study highlights the importance of microbiome biogeography between populations despite relatively small ecological differences between each population's respective environment. Further, we encourage future consideration and quantification of the importance of microbiomes during translocations of individuals, even between genetically compatible populations from similar ecotypes (Wang et al., 2019).

Limited interaction between animals from isolated populations may also contribute to phylogenetic divergence between populations by limiting the homogenizing effects of species dispersal thus increasing genetic drift (Moeller et al., 2017). Conversely, within socially structured populations or close‐kin, the increased transmission of microbes can create strong local patterns of microbial diversity (Blaser, 2015; Tesson et al., 2015). Ad‐hoc support for limited transmission between the sampled elk populations can be found in a study by Hand et al. (2014), who described limited female‐specific gene flow (mitochondrial *F*_ST_ = 0.161) in 23 elk populations near our study area. Thus, in our system, microbiome patterns may be maintained by limited female movement and geographic distance, but this remains to be tested and may not hold across all elk populations. Seasonal variation in microbiomes is also well‐documented (Amato et al., 2015; Maurice et al., 2015; Ren et al., 2017), and annual variation also seems likely, but the importance of these factors remains unknown in elk. Microbiome structure between elk populations is likely not due to host genetic differences since low overall genetic diversity (nuclear *F*_ST_ = 0.002) has also been reported between populations of elk from the study area (Hand et al., 2014). Ultimately, a number of these and other factors may be involved in structuring microbiome composition in the populations we sampled and might explain the increased microbiome variation we observed in the Tobacco‐root elk population.

The results of the LDA models tested support a strong biological connection between fecal microbiomes and elk sex, body‐fat, and age. Classifier accuracies for sex and body‐fat were high, despite combining data from multiple elk populations that, as discussed, included the strong (and potentially confounding) signal of biogeographic diversity in the total microbiome. Our positive model results are unique compared to some other studies attempting to associate the microbiomes of host animals to body condition, sex, and age (Bennett et al., 2016; Fountain‐Jones et al., 2020; Mshelia et al., 2018a). For example, previous studies found either no significant connection between the microbiome, host age, social group, and environment (Bennett et al., 2016), or incomplete support (Fountain‐Jones et al., 2020; Mshelia et al., 2018a; Ren et al., 2017). Substantive differences between our study methods and bioinformatic approaches likely contributed to these seemingly contradictory outcomes. These different approaches and conclusions illustrate the importance of applying various mechanistically diverse methods and their continued development for host‐microbiome linkages.

Consistent with our findings, a culture‐based study of gut microbiomes in horses (*Equus caballus*) found significant associations with sex, age, and body‐condition scores (Mshelia et al., 2018b). This study provides promising corroborative results to our own, despite the horse study being based on culture‐dependent microbes and the physiological differences between horses and elk (hind‐gut fermenter vs ungulate). Our combined findings also strengthen arguments for the holobiont theory, which suggests a fundamental evolutionary relationship between the microbiome and many host factors (Carrier & Reitzel, 2017; Zilber‐Rosenberg & Rosenberg, 2008).

Sex‐specific microbiomes were found in the elk sampled in this study based on both FS‐LDA sex classifier results and significant Adonis tests of beta‐diversity when sex was used as a covariate (Bray‐Curtis, *p* = .002; weighted‐Unifrac, *p* = .018). Thus, there is value in examining sex‐specific differences in microbiomes, since these differences may affect the success of conservation‐related microbiome augmentation in captive populations (Haworth et al., 2019). Also of relevance, since ungulate (and other species) population management requires diverse outcomes, monitoring population‐specific sex ratios can help inform the management action (Toïgo & Gaillard, 2003). This is especially pertinent because adult female elk survival is typically the best predictor of future population growth rate (Gaillard et al., 2000). It is therefore useful to identify sex via non‐invasive fecal sampling. Although alternatives for sex determination exist (e.g., SRY gene PCR) and significant hurdles remain to the application of microbiome‐based estimates of sex‐ratios (e.g., randomized sampling schema) it remains a promising possibility.

Estimates of animal body‐fat and age are both important for elk population management (J. G. Cook et al., 2016). Female body condition is a good predictor of pregnancy, which becomes population growth rate (Morano et al., 2013) and differences in fertility of female ungulates between age classes are most often caused by age‐specific variation in body condition (Albon et al., 1983; Cook et al., 2004; DelGiudice et al., 2007; Ropstad, 2000). We found evidence for an association between elk body‐fat and the microbiome using three models of the FS‐LDA classifier (2‐, 3‐, or 4‐class models with 91%, 77%, and 58.3% CV accuracy, respectively). Specific bacterial taxa associated with elk body‐fat were similar across all three models tested (e.g., Bacteroides, Butyricimonas, Clostridium_XIVa, Grancilibacter, and Tannerella; Table S3). This provides support for those taxa having a role in the functional phenomena behind body‐fat measurement, although such experiments were beyond the scope of this study.

We also demonstrated that adult female elk age differences can be detected in the microbiome using a 2‐class model. Age‐related differences in the microbiome have been demonstrated in humans and other model animals (Lan et al., 2013; Xing et al., 2020), but evidence in wildlife is still emerging (Xing et al., 2020). Adult age classes undergo microbial succession in response to diverse physiological and environmental forces (Lan et al., 2013; Stephens et al., 2016). Our study provides additional support for the role of adult successional change using the elk bacterial microbiome. Changes in diet and corresponding microbial succession during early transitional development are somewhat more obvious in mammals (like elk calves) that initially consume mothers’ milk, then change to plant foraging (Blaser, 2015; Koenig et al., 2011). A limitation of our elk age model is the incomplete representation of bookend classes including calves and older elk. Although we did not sample elk calves, the diet transition from milk to forage and the physiological development of the rumen chambers both suggest a dramatic change in microflora occurs. Once the 0–1 age‐class is included in the model, we expect that a non‐invasive survey technique may be possible to support further investigation of age‐related population dynamics that improve management in hard to observe elk populations.

## 5 CONCLUSION

Our results show that elk microbiomes respond to both the strong extrinsic factor of biogeography and simultaneously occurring, but more subtle, intrinsic forces of individual body‐fat, sex, and age‐class. Thus, we have developed and described herein an approach that allows us to disentangle microbiome responses attributed to multiple factors of varying strength from the same bacterial microbiome sequence data set. In future cases, once strongly associating microbial taxa are vetted for stability among populations in space and time, analysis of fecal microbiome biomarkers may represent a less invasive alternative for acquiring information on wildlife populations than traditional sampling methodologies. Understanding the seasonal stability of the microbiome and the reproducibility of our FS‐LDA models in these and other elk populations would be prudent before applying the results and methods of this study. Nonetheless, the research approach and bioinformatic tools reported here provide a foundation for the continued development of microbiome associations in elk for future monitoring and conservation. We are hopeful that these methods can be expanded to investigate a diverse range of wildlife species (including non‐mammals) that have strong host‐microbiome mutualism. Additionally, our findings across multiple host factors from the same fecal microbiome dataset help unite some formerly unconfirmed expectations from host‐microbiome theory regarding the diverse interconnections of microbiomes and hosts. The products of such efforts could eventually provide insights and novel solutions to current wildlife management issues and allow threatened and endangered species to be studied with less perturbation.

## CONFLICT OF INTEREST

The authors declare no conflict of interest.

## AUTHOR CONTRIBUTIONS

**Samuel B. Pannoni:** Conceptualization (equal); Data curation (lead); Formal analysis (lead); Funding acquisition (lead); Investigation (lead); Methodology (equal); Project administration (supporting); Resources (lead); Software (lead); Supervision (equal); Validation (lead); Visualization (lead); Writing – original draft (lead); Writing – review & editing (lead). **Kelly M. Proffitt:** Conceptualization (equal); Investigation (equal); Methodology (equal); Project administration (equal); Resources (equal); Supervision (equal); Writing – review & editing (supporting). **William E. Holben:** Conceptualization (equal); Formal analysis (supporting); Funding acquisition (supporting); Methodology (supporting); Project administration (supporting); Resources (equal); Supervision (supporting); Writing – original draft (supporting); Writing – review & editing (supporting).

## Supporting information

Appendix S1

## Data Availability

The raw sequence dataset and metadata is deposited in the Dryad repository under accession number https://doi.org/10.5061/dryad.4j0zpc880. R scripts used to process sequences and build classifiers are available upon request.
