# Supplementary material for: Non‐invasive monitoring of multiple wildlife health factors by fecal microbiome analysis
Source: Ecol Evol. 2022 Feb 9;12(2):e8564. doi: 10.1002/ece3.8564 (PMC8826075; doi:10.1002/ece3.8564)
Supplement: Supplementary file 1 — Appendix S1 [file ECE3-12-e8564-s001.docx]

**Supplemental Information for:**

**Non-invasive Monitoring of Multiple Wildlife Health Factors by Fecal Microbiome Analysis.**

**Samuel B. Pannoni, Kelly M. Proffitt and William E. Holben**

**Supplemental Figure 1 - Cross-validation box and whisker plots for population classifier performance**


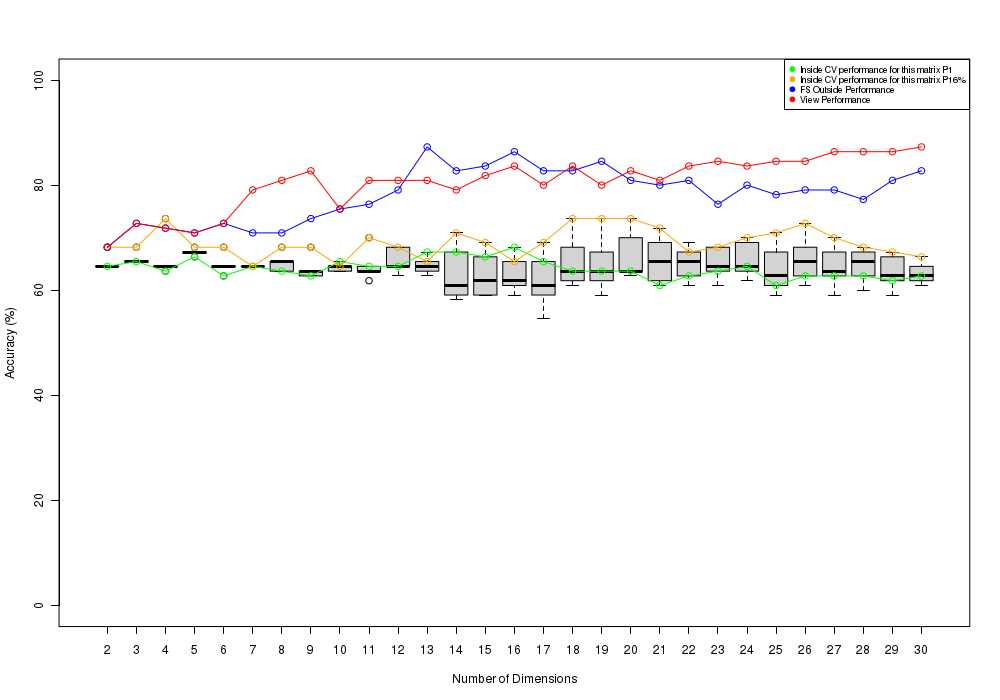


Box plots of cross-validation (CV) accuracies (y-axis) with standard error whiskers from 4 types of CV approaches selecting between 1 and 30 dimensions (x-axis). Female elk microbiome data were combined from 4 populations in Montana, then feature selected by population. The green line indicates inside CV performance approach; the yellow line is inside CV performance for a zero reduced OTU matrix; the blue line is feature selection (FS) conducted outside of CV, and the red line is the performance for the LDA plots. Grey box and whiskers represent the mean and quartiles of model accuracy at each feature number using progressively zero pruned data sets from 0% - 16. This plot helps determine the optimal number of features to balance accuracy, variance and reduce over-fitting of the algorithm (the 23-dimension dataset is plotted and presented in the manuscript as Figure 4). This figure was produced in R.

**Supplemental Figure 2 - Microbiome bar-plots of bacterial genera per elk**

Stacked bar plots of microbiome members proportional abundance calculated for all elk sampled (N=110; including 6 elk of unknown sex). Bacterial genera are indicated by colors in the legend, with grey indicating an unsuccessful taxonomic classification at the genus level and magenta indicating all bacteria per animal with a proportional abundance <1%. Plot produced in R using the Phyloseq package.

**Supplemental Figure 3 - Breakaway richness estimation of elk microbiomes**

Breakaway method richness estimation plot of elk microbiomes from 4 populations. Colored circles represent individual elk richness estimates with error bars representing modeled estimates of true (unmeasured but present) richness.

**Supplemental Figure 4 - Breakaway Shannon sample diversity for elk microbiomes**

Breakaway method Shannon diversity (SD) estimation plot of elk microbiomes from 4 populations. Colored circles represent individual elk microbiome diversity estimates with error bars representing modeled estimates of true (unmeasured but present) SD.

**Supplemental Figure 5 - Beta-diversity ordination plots**

Ordination plots of elk microbiome beta-diversity using: A) weighted-Unifrac distance with PCoA, B) unweighted Unifrac distance with PCoA and C) Bray-Curtis distance with RDA. Populations are represented by four colors and elk sex is indicated by shape.

**Supplemental Table 1.**

A summary of elk field measurements by factors of age, sex, population and ingesta-free body-fat (IFBF) percentage.

| Field Measurements | | |
| --- | --- | --- |
| Age |  |  |
|  | min | 3 |
|  | max | 10 |
|  | mean (SD) | 6.38 (2) |
| Sex |  |  |
|  | F | 87 |
|  | M | 17 |
|  | Unknown | 6 |
| Population |  |  |
|  | Bitterroot | 13 |
|  | Tobacco-Root | 18 |
|  | Blacks-Ford | 25 |
|  | Sapphire | 54 |
| IFBF |  |  |
|  | min | 5.69 |
|  | max | 13.29 |
|  | mean (SD) | 7.95 (1.36) |
|  | Unknown | 37 |

**Supplemental Table 2.**

Summary of sequence and ASV filtering across samples.

| Per sample | Min | Mean | Max | Total |
| --- | --- | --- | --- | --- |
| Sequences | 5,571 | 79,770 | 128,069 | 8,774,731 |
| Filtered seqs. | 1,103 | 30,786 | 66,124 | 3,386,467 |
| ASVs post-filter | 1,008 | 30,736 | 65,894 | - |
| Observed Richness | 30 | 714.8 | 1,290 | 11,957 |

**Supplemental Table 3.**

Bacterial genera used in each FS-LDA classifier. Each classifier selected a subset of the available features (bacteria) that optimized classification performance for each factor (columns). These bacteria are provided for inferring possible underlying functional relevance and overlap between classifiers groups.

| **Population** | **IFBF (2 groups)** | **IFBF (3 groups)** | **IFBF (4 groups)** | **Sex (Boot) (2)** | **Sex (Rarefied) (1)** | **Age (2 groups)** |
| --- | --- | --- | --- | --- | --- | --- |
| Acetanaerobacterium | Anaerobacterium | Acetivibrio | Alloprevotella | Aestuariispira | Aestuariispira | Anaerofustis |
| Acetitomaculum | Anaerofustis | Adlercreutzia | Anaerofustis | Akkermansia | Alloprevotella |  |
| Adlercreutzia | Anaeroplasma | Alistipes |  | Anaerorhabdus | Anaerofilum |  |
| Aestuariispira | Anaerorhabdus | Anaerostipes | Anaerostipes | Anaerostipes |  |  |
| Anaerobacterium | Anaerovorax | Anaerotruncus |  | Anaerovorax |  |  |
| Anaerovorax |  |  |  |  |  |  |
| Bilophila | Bacteroides | Bacteroides | Butyricimonas | Bacteroides | Bacteroides |  |
| Blautia |  | Bilophila |  | Barnesiella |  |  |
| Butyricicoccus |  | Butyricicoccus |  |  |  |  |
| Cerasicoccus |  | Christensenella | Cerasicoccus | Clostridium_XlVb | Clostridium_XlVb | Clostridium_XVIII |
| Clostridium_III | Clostridium_XlVa | Clostridium_XlVa | Clostridium_XlVa | Coraliomargarita |  | Coraliomargarita |
|  |  | Clostridium_XlVb | Coprobacillus |  |  |  |
|  |  | Coprobacillus | Coprobacter |  |  |  |
|  |  |  | Dehalobacter |  | Dehalobacter |  |
| Eubacterium | Elusimicrobium | Eisenbergiella | Ethanoligenens | Ercella |  |  |
|  | Eubacterium | Escherichia/Shigella | Fibrobacter | Ethanoligenens |  |  |
| Faecalitalea | Faecalitalea |  |  | Faecalibacterium | Faecalibacterium | Flavonifractor |
| Friedmanniella | Fibrobacter |  |  | Flavonifractor |  |  |
|  | Flavonifractor |  |  |  |  |  |
|  | Gracilibacter | Gracilibacter | Gracilibacter |  |  |  |
|  |  |  | Hungatella |  |  | Hungatella |
| Intestinimonas | Intestinibacter | Intestinibacter | Intestinimonas |  |  |  |
|  |  |  |  | Lactobacillus |  |  |
|  |  |  |  | Lutispora |  |  |
| Mucinivorans | Methanimicrococcus | Methanimicrococcus |  | Methanomassiliicoccus | Methanomassiliicoccus |  |
|  | Mucinivorans |  |  |  |  |  |
|  | Mucispirillum |  |  |  |  |  |
| Ornatilinea | Ornatilinea | Odoribacter | Oxalobacter | Ornatilinea | Odoribacter |  |
|  | Oxobacter | Oscillibacter | Oxobacter |  |  |  |
| Paludibacter | Paludibacter | Paraprevotella |  | Parabacteroides |  |  |
|  | Parasutterella | Peptococcus | Peptococcus |  |  |  |
|  | Phocaeicola | Phocaeicola | Phascolarctobacterium |  |  |  |
|  | Prevotella |  | Pseudobacteroides |  |  |  |
|  | Pseudobacteroides |  | Pseudoflavonifractor |  |  |  |
| Roseburia | Rubinisphaera |  | Rikenella | Rikenella |  |  |
|  |  |  | Robinsoniella | Robinsoniella | Robinsoniella |  |
|  | Ruminococcus | Ruminococcus | Roseburia | Ruminococcus2 |  |  |
| Saccharopolyspora |  | Sporobacter | Slackia | Saccharofermentans |  | Saccharofermentans |
| Slackia |  | Staphylococcus | Sporobacterium | Sphaerochaeta | Sphaerochaeta | Sporobacter |
| Sporobacter |  | Streptococcus | Streptococcus | Staphylococcus |  | Syntrophococcus |
|  |  | Succinivibrio |  |  |  |  |
|  |  | Sutterella |  |  |  |  |
|  | Tannerella | Tannerella | Tannerella |  |  |  |
|  | Turicibacter | Turicibacter |  |  |  |  |
| Vampirovibrio |  |  | Vampirovibrio |  |  |  |
